# Biochemical approach for isolation of polyadenylated RNAs with bound proteins from yeast

**DOI:** 10.1016/j.xpro.2021.100929

**Published:** 2021-11-01

**Authors:** Ana M. Matia-González, Ibtissam Jabre, André P. Gerber

**Affiliations:** 1 Department of Microbial Sciences, School of Biosciences and Medicine, Faculty of Health and Medical Sciences, University of Surrey, Guildford, Surrey GU2 7XH, UK

**Keywords:** Model Organisms, Molecular Biology, Gene Expression, Protein Biochemistry

## Abstract

*In vivo* characterization of RNA-protein interactions is the key for understanding RNA regulatory mechanisms. Herein, we describe a protocol for detection of proteins interacting with polyadenylated RNAs in the yeast *Saccharomyces cerevisiae.* Proteins are crosslinked to nucleic acids *in vivo* by ultraviolet (UV) irradiation of cells, and poly(A)-containing RNAs with bound proteins are isolated from cell lysates using oligo[dT]_25_ beads. RBPs can be detected by immunoblot analysis or with mass spectrometry to define the mRNA-binding proteome (mRBPome) and its changes under stress.

For complete details on the use and execution of this protocol, please refer to Matia-González et al. (2021, 2015).

## Highlights

• Detection of proteins that interact with polyadenylated RNAs *in vivo*
• UV irradiation of cells to crosslink RNA-protein interactions
• Polyadenylated RNAs are captured with oligo[dT] beads from yeast cell lysate
• Suitable for monitoring dynamic changes of the mRNA-binding proteome

## Graphical abstract

**Figure:**
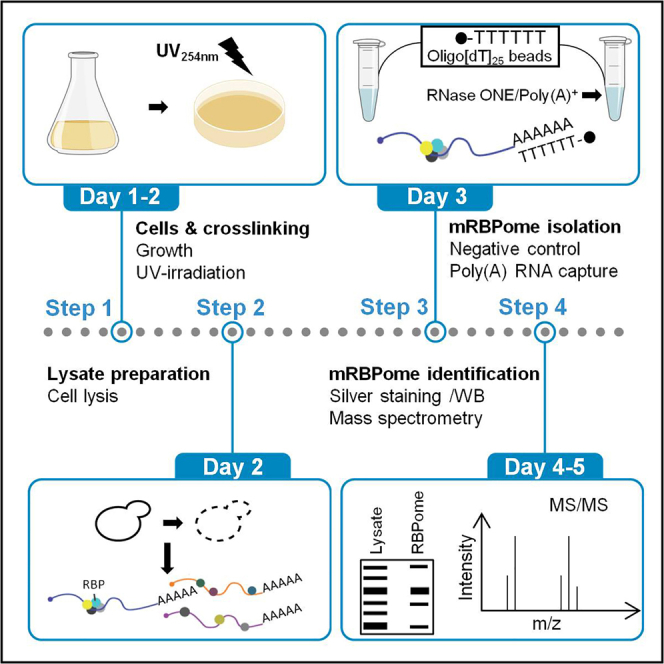

## Before you begin

The protocol describes the specific steps for poly(A) RNA interactome capture (RIC) from UV-irradiated budding yeast cells grown in rich media (Yeast-Peptone-Dextrose (YPD)). The protocol has also been applied to cells treated with 0.5 mM hydrogen peroxide (H_2_O_2_) as a stress reagent for induction of mild-oxidative stress (Matia-González et al., 2021).

The procedure is based on the capture of poly(A) RNA, a major component of it comprising mRNAs. Hence, the protocol presented here is preferred for investigation of mRNA regulation executed by mRNA binding proteins (mRBPs) that mediate the processing of mRNA precursors (pre-mRNAs) in the nucleus, the export and localization of mRNAs to different subcellular locations in the cytoplasm, and the translation and eventual decay of mRNAs (Singh et al., 2015). We wish to note that there are alternative RIC protocols aimed at isolation of proteins associated with total RNA from yeast (including highly abundant ribosomal RNAs, tRNAs) and are based on organic phase extraction (Queiroz et al., 2019) or the use of silica beads (Shchepachev et al., 2019). In any cases, cells are exposed to UV-irradiation to promote covalent crosslinks between the RNA and proteins in close proximity. However, it should be noted that UV-irradiation of cells can also lead to protein-protein and protein-DNA crosslink, a possibility to be considered for interpretation of the data.

Yeast lysates contain a substantial amount of enzymatic activities (e.g., RNases, DNases, and proteases). Cell lysates and samples should therefore be kept on ice, which substantially reduces enzymatic activity. It is also necessary to add RNase and protease inhibitors to buffers/samples immediately before use according to the suggestions given in the protocol. Furthermore, we recommend using sterile filter tips and RNase-free tubes with low RNA/protein binding capacities. Gloves must be worn, and the protocol should be performed in an RNase-free workspace, which can be accomplished by cleaning surfaces with RNase ZAP^TM^ or likewise RNase decontamination solutions.

### Media preparation and streaking of yeast cells on agar plates

**Timing: 2–3 days**

Before starting the protocol, sterile media needs to be prepared for the growth of yeast *Saccharomyces cerevisiae* (*S. cerevisiae)* cells. In our case, we used autoclaved YPD media, a rich media supplemented with glucose as the carbon source (Sherman, 2002). The desired yeast strain needs then to be streaked on YPD plates so then, a single colony can be picked for growth in liquid culture later on.

1. Create a sterile environment by turning on a Bunsen burner.

***Note:*** The updraft created by the flame for sterility works best within a radius of 30–70 cm.

2. Remove approximately 10–20 μL *S. cerevisiae* cells (e.g., strain BY4741) from the glycerol stock and streak cells on a YPD agar plate with a sterile inoculation loop.

***Note:*** Keep the glycerol stock on ice to prevent rapid defrosting. Put the stock vial back to the −80°C freezer immediately after use.

***Note:*** We used the BY4741 strain, but the protocol is also applicable to other *S. cerevisiae* strains.

3. Incubate the plate upside-down in a 30°C incubator in the dark until individual colonies have approximately 0.2–0.3 cm diameter (usually about 2 days).

***Note:*** The plate can be stored at 4°C for up to 3 months.

## Key resources table

**Table:** 

REAGENT or RESOURCE	SOURCE	IDENTIFIER
**Antibodies**		

Mouse anti-Pab1 (1:5,000)	antibodies-online GmbH	Cat#ABIN1580454
Mouse anti-Pgk1 - clone 22C5D8 (1:5,000)	AbCam	Cat#ab113687; RRID:AB_10861977
Mouse anti-Act1 (1:2,500)	MP Biomedicals	Cat#0869100; RRID:AB_2335304
PAP reagent (1:5,000)	Sigma	Cat#P1291; RRID:AB_1079562
HRP-conjugated sheep anti-mouse IgG (1:5,000)	Amersham	Cat#NA931; RRID:AB_772210

**Chemicals, peptides, and recombinant proteins**		

cOmplete EDTA-free protease inhibitors	Roche	11873580001
RNasin® Ribonuclease inhibitor	Promega	N2511
Polyadenylic acid potassium salt (Poly(A))	Sigma	P9403
RNase ONE	Promega	M4265
peqGREEN RNA/DNA dye	Peqlab	732–3196
Precision Plus Protein Dual Color Standards	BIO-RAD	1610374
Tris	Thermo Fisher	75825
HCl	Sigma	320331
Lithium chloride	Acros Organics	413275000
EDTA	Sigma	E5134
Triton X-100	Acros Organics	327372500
DTT	Sigma	43816
Tween-20	BIO-RAD	1706531
Skim milk powder	Sigma	70166
Yeast Extract	Oxoid	LP0021
Peptone	Oxoid	LP0037
Dextrose	Sigma	D9434
Agar	Oxoid	LP0028

**Critical commercial assays**		

ZR RNA MiniPrep kit	Zymo Research	R1065
Dynabeads™ mRNA DIRECT™ Purification Kit	Life Technologies	61011
Transcriptor High Fidelity cDNA Synthesis Kit	Roche	05091284001
ProteoSilver™ Plus Silver Stain Kit	Sigma-Aldrich	PROTSIL2-1KT

**Experimental models:Organisms/strains**		

*Saccharomyces cerevisiae* strain BY4741: MAT**a***his3*Δ*1 leu2*Δ*0 met15*Δ*0 ura3*Δ*0*	Euroscarf collection	Y00000

**Oligonucleotides**		

ACT1_Fw,5′-GTCTGGATTGGTGGTTCTATC-3′	This lab	N/A
ACT1_Rev, 5′-GGACCACTTTCGTCGTATTC-3′	This lab	N/A

**Other**		

Polyvinylidene difluoride (PVDF) membranes	Thermo Fisher	88518
Microcon-10 kD Centrifugal Filter Unit with Ultracel-10 membrane	Millipore	MRCPRT010
4%–15% Mini-PROTEAN® TGX™ Precast Protein Gels	BIO-RAD	4561083
0.22 μm EMD Millipore Steriflip	Millipore	SCGP00525
Disposable Sterile Filter Systems	Corning	431097
Dyna-Mag-2 Magnet	Invitrogen	12321D
Eppendorf Protein LoBind microcentrifuge tubes	Sigma	Z666505
50 mL Corning Centrifuge Tubes	Corning	CLS430291
15 mL Corning Centrifuge Tubes	Corning	CLS430791
Disposable Inoculation Loop, 10 μL	Fischer Scientific	12870155
Quick Start™ Bradford 1**×** Dye Reagent	BIO-RAD	5000205
Ponceau-S	Sigma	P3504
Amicon® Ultra-4 Centrifugal Filter Unit	Millipore	UFC8100
RNase ZAP^TM^ RNase Decontamination Solution	Thermo Fisher	AM9780
RNase/DNase free Safe-lock 2 mL microcentrifuge tubes	Thermo Fisher	10031282
15 cm Petri Dish	Thermo Fisher	P5981
Glass beads 0.5 mm	Biospec	11079105
Disposable plastic cuvettes for spectrophotometer	Delle industrie	01938–00
Western blot filter paper	Thermo Fisher	84783
Immobilon® Western Chemiluminescent HRP Substrate	Immobilon	WBKLS0500
Analog (SHKA-) MaxQTM Floor Shaker Incubator	Thermo Fisher	4358
3L Erlenmeyer flasks	Fischer Brand	FB33136
100 mL Erlenmeyer flasks	Fischer Brand	FB33131
Spectrophotometer (UV/Vis single holder)	Jenway	6715
The Stratalinker® UV crosslinker	Stratagene	1800
TissueLyser II	Qiagen	RETSCH MM200
TissueLyser Adapter Set 2 **×** 24	Qiagen	69982
Refrigerated centrifuge	Eppendorf	5804R
Swing-bucket rotor	Eppendorf	A-4-44
Fixed angle rotor	Eppendorf	FA-45-30-11
NanoDrop2000 Microvolume Spectrophotometer	Thermo Fisher	ND-2000
Trans-Blot® Turbo™ Transfer System	BIO-RAD	1704150

## Materials and equipment

### Yeast media

***Note:*** Distilled (d) water (H_2_O) was used to prepare yeast media.

***Note:*** Yeast media must be autoclaved before use for 20 minutes at 121°C.

**Table:** 

YPD media		
Reagent	Final concentration	Amount
Yeast Extract	1%	10 g
Peptone	2%	20 g
Dextrose	2%	20 g
dH_2_O	n/a	1,000 mL
**Total**	**n/a**	**1,000 mL**

To prepare the media, yeast extract and peptone (YEP) is dissolved in 900 mL of dH_2_O and autoclaved. To avoid the Maillard reaction (a chemical reaction between amino acid and reducing sugar that will change the medium into a brownish color, dropping the actual sugar concentration (Wang and Hsiao, 1995)), it is recommended to dissolve the 20 g dextrose separately in 100 mL dH_2_O and sterile filter (0.22 μm). 100 mL of the sterile 20% dextrose solution is finally added to 900 mL YEP under sterile conditions, obtaining 1 L of YPD media.

***Note:*** Media and dextrose stock solutions can be stored for up to one year at room temperature (22°C–25°C).

**Table:** 

YPD agar		
Reagent	Final concentration	Amount
Yeast Extract	1%	10 g
Peptone	2%	20 g
Dextrose	2%	20 g
Agar	2%	20 g
dH_2_O	n/a	1,000 mL
**Total**	**n/a**	**1,000 mL**

Autoclave ingredients without dextrose in 900 mL dH_2_O in a 1 L glass bottle. After autoclaving, add 100 mL of 20% sterile dextrose solution. Keep the bottle with YPD agar at least at 55°C in a water-bath to avoid media solidification. Pour plates under sterile conditions and prevent the formation of air bubbles (approximately 20 mL of YPD agar per 10 cm standard dish). Solid YPD plates must be stored at 4°C and protected from light.

***Note:*** Pour the plates in a fume hood or in the vicinity of a Bunsen burner flame. The flame can also be used to remove air-bubbles from the surface.

### Buffers for poly(A) RIC

***Note:*** Double-distilled (dd) RNase-free water (H_2_O) was used through the procedure.

***Note:*** Buffers are preferentially prepared freshly from sterile stock solutions and filtered (0.22 μm).

**Table:** 

Lysis buffer		
Reagent	Final concentration	Amount
Tris-HCl, pH 7.5 (1 M)	100 mM	1 mL
LiCl (3 M)	500 mM	1.66 mL
EDTA (0.5 M)	10 mM	0.2 mL
Triton X-100 (20%)	1%	0.5 mL
DTT (1 M)	5 mM	0.05 mL
RNasin (40 U/μL)	100 U/mL	0.025 mL
Complete EDTA-free protease-inhibitor cocktail (10×)	1×	1 tablet
ddH_2_O	n/a	6.365 mL
**Total**	**n/a**	**10 mL**

Buffer can be stored at 4°C for up to 1 day without inhibitors. Inhibitors must be freshly added before use.

**Table:** 

Wash buffer A		
Reagent	Final concentration	Amount
Tris-HCl, pH 7.5 (1 M)	10 mM	0.1 mL
LiCl (3 M)	600 mM	2 mL
EDTA (0.5 M)	1 mM	0.02 mL
Triton X-100 (20%)	0.1%	0.05 mL
ddH_2_O	n/a	7.83 mL
**Total**	**n/a**	**10 mL**

Buffer can be stored at room temperature for up to 1 month.

***Note:*** Wash buffer A can be supplemented with 10 U/mL of RNasin if RNA degradation is of concern (troubleshooting problem 1). Add 0.2% lithium dodecyl sulphate (LiDS) to increase stringency if troubleshooting problem 3 is of concern.

**Table:** 

Wash buffer B		
Reagent	Final concentration	Amount
Tris-HCl, pH 7.5 (1 M)	10 mM	0.2 mL
LiCl (3 M)	600 mM	4 mL
EDTA (0.5 M)	1 mM	0.04 mL
ddH_2_O	n/a	15.76 mL
**Total**	**n/a**	**20 mL**

Buffer can be stored at room temperature for up to 1 month.

**Table:** 

Elution buffer		
Reagent	Final concentration	Amount
Tris-HCl, pH 7.5 (1 M)	10 mM	0.2 mL
ddH_2_O	n/a	9.8 mL
**Total**	**n/a**	**10 mL**

Buffer can be stored at room temperature for up to 1 month.

## Step-by-step method details

### Cell culture and UV irradiation

**Timing: 1.5 days**

In this step, the cells are UV-irradiated to crosslink RNA-protein interactions *in vivo*, enabling the subsequent capture of both permanent and transient binding proteins. A sample with non-irradiated cells can be included to control the crosslinking. Other controls, such as the poly(A) competition used in this protocol are introduced in the next step of the protocol.

1. Pick 1 yeast colony from the YPD agar plate with a sterile loop and inoculate 20 mL YPD media placed in a sterile 100 mL Erlenmeyer flask. Place the flask in a shaker and grow cells overnight (12–14 h) at 30°C with constant shaking at 220 rounds per minute (r.p.m.).

***Note:*** To ensure good aeration of cells, we recommend using at least 1:5 ratio of culture volume to Erlenmeyer flask volume (i.e., 20 mL of culture in a 100 mL Erlenmeyer flask).

2. Refresh culture in 500 mL YPD placed in a sterile 3 L Erlenmeyer flask, setting OD_600_ ∼0.1, and grow at 30°C at 220 r.p.m to OD_600_ ∼0.6. This takes about 3 h 30 min (doubling time of cells ∼90 min).
  a. Set the spectrophotometer at 600 nm wavelength.
  b. Set up a blank cuvette by placing 1 mL YPD in a disposable plastic cuvette.
  c. Place the blank cuvette in the spectrophotometer and take the blank measurement (absorbance (A) = 0.00).
  d. Make a 1:10 dilution of the 20 mL pre-culture and measure the absorbance.
  e. Calculate the volume of pre-culture required to inoculate 500 mL YPD with a starting OD_600_ ∼0.1.
  f. Remove appropriate volume of pre-culture with a pipette and transfer cells to the 500 mL YPD in a sterile 3 L Erlenmeyer flask.
  g. Place the culture in a shaker and grow cells at 30°C at 220 r.p.m. Measure the OD_600_ every 2 h until the OD_600_ reaches ∼0.4. At this point, cells have passed the lag-phase and reach mid-log phase, so it is advisable to start the measures every 30 min.

***Note:*** Cells can also be subjected to stress treatments to eventually monitor changes in RNA-protein associations as compared to untreated cells. For instance, we treated yeast cells at mid-log phase (i.e. OD_600_ ∼0.6) with 0.5 mM hydrogen peroxide (H_2_O_2_) for 15 min to induce a mild oxidative stress response (Matia-González et al*.,* 2021). However, it is essential to confirm that an appropriate stress response has been induced, which can be assessed by monitoring the expression of stress-specific markers.

3. Irradiate cells with UV light at 254 nm to crosslink RNA-protein interactions.
  a. Harvest cells in 500 mL centrifuge tubes at OD_600_ ∼0.6 by centrifugation at 3,000 × *g* in a swing-bucket rotor for 3 min at room temperature (22°C–25°C).
  b. Wash cells three times at room temperature with 25 mL phosphate-buffered-saline (PBS). Collect cells in between by centrifugation as indicated in the previous step.
  c. Resuspend cells in 15 mL 1× PBS.
  d. Spread the cell-suspension in a 15 cm petri dish.
  e. Place the dish on ice in the UV-crosslinker. Expose cells to 3 × 400 mJ/cm^2^ of 254 nm UV-light with two 2-min breaks and gentle mixing between each exposure.
  f. Harvest cells by centrifugation at 3,000 × *g* for 3 min at 4°C.
4. Snap freeze pellets in liquid nitrogen.

**Pause point:** Cell pellets can be stored at −80°C for up to two months.

**CRITICAL:** It is important that cells are evenly spread in the dish, making a thin layer of cells for exposure to UV-light.

### Lysate preparation

**Timing: 1 h 30 min**

Cell lysates are prepared by mechanical disruption of cells with glass beads in a TissueLyser.

5. Resuspend cells in 4 mL lysis buffer by pipetting up and down on ice and split cells in 4 × 2 mL safe-lock microcentrifuge tubes.
6. Add 2/3 vol. glass beads to each tube.
7. Place the tubes balanced in a TissueLyser adapter and break cells at 30 Hz for 10 min at 4°C.

***Note:*** The TissueLyser is preferentially placed in the cold room to maintain samples at 4°C whilst lysis. Tubes must be balanced within the TissueLyser adapter set to avoid motor failures.

8. Clear lysate by three sequential centrifugations at 3,000 × *g* for 3 min, and 5,000 × *g* and 10,000 × *g* for 5 min each at 4°C. Carefully remove the supernatant with a filter tip and transfer to a fresh microcentrifuge tube after each centrifugation, avoiding any pellet contamination.

***Note:*** Repeated centrifugation at progressively higher-speed removes non-lysed whole cells (3,000 × *g*), cell debris (5,000 × *g*) and nuclei and cytoskeletons (10,000 × *g*).

9. After the last centrifugation, combine all supernatants in a 15 mL Corning centrifuge tube.
10. Quantify protein concentration of the final extract with a Bradford assay taking bovine serum albumin (BSA) as a reference standard following the manufacturer’s guidelines.

***Note:*** Typically, we obtained a concentration of ∼3.5 mg/mL protein.

11. Concentrate protein sample up to 10 mg/mL with an Amicon® Ultra-4 Centrifugal Filter Unit 10 kDa, following the manufacturer’s guidelines. Ensure that the final volume does not go below 1.2 mL. Prepare 500 μL aliquots containing ∼5 mg of protein lysate (∼10 mg/mL) in Eppendorf Protein LoBind microcentrifuge tubes.

***Note:*** Make sure the membrane of the Centrifugal Unit is not getting damaged with the pipette tip. Quantify protein amount after concentration as explained in step 10 and ensure that proteins have not aberrantly leaked through a damaged membrane.

12. Keep 75 μL of the extract as reference. 50 μL will be used to assess the RNA quality and the remaining can be used for protein analysis (see below).

**Pause point:** Lysates can be kept on ice and immediately used for later steps in the protocol, or snap frozen in liquid nitrogen and stored at −80°C for up to one month.

**Control step**: Analysis of RNA integrity before proceeding with poly(A) RIC.

13. RNA quality control.
  a. Isolate total RNA from 50 μL of extract (=input) with the ZR RNA MiniPrep kit, following manufacturer’s guidelines.
  b. Quantify RNA with a Nanodrop ND-2000 device.
  c. Visualize 1 μg RNA on a 1% agarose gel stained with peqGREEN RNA/DNA dye.

***Note:*** Typically, we obtained 30 μg of total RNA from 50 μL extract.

**Figure 1:**
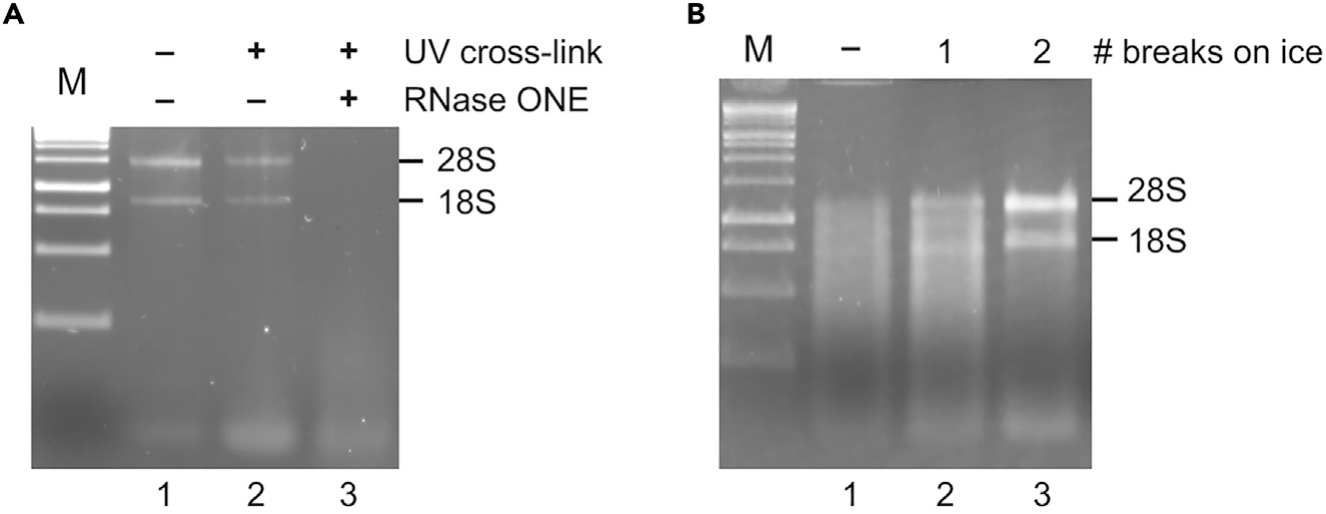
RNA analysis after UV-irradiation of cells (A) Testing UV-irradiation and RNase control digest. One μg of total RNA was electrophoresed on a 1% agarose gel and stained with peqGREEN DNA/RNA dye. Lane 1: RNA from non-irradiated cells; Lane 2: RNA from UV-irradiated cells. Lane 3: RNA from UV-irradiated cells and RNase ONE treated extracts. M: molecular weight marker. (B) RNA degradation is prevented by UV-irradiation on ice. Lane 1: RNA after exposure of cells to 1,200 mJ without any breaks. Lane 2: one break on ice for 2 min after 600 mJ. Lane 3: 2 breaks on ice (after 400 mJ). M: molecular weight marker.

**CRITICAL:** We highly recommend checking RNA integrity after UV-irradiation by comparing the pattern with a non-crosslinked sample. Ribosomal RNA (rRNA) bands should be clearly visible before proceeding with the poly(A) RNA isolation (Figure 1A, lanes 1 and 2). Troubleshooting problem 1.

In addition, when RNase ONE is included as negative control, RNA integrity after digestion must be analyzed to ensure that RNA is completely degraded and rRNA species cannot be distinguished and only a smear can be visualized (Figure 1A, lane 3).

***Note:*** Total RNA can be stored at −80°C for further analysis.

### Poly(A) RNA interactome capture

This step describes the recovery of poly(A) RNA and interacting proteins with oligo[dT]_25_ magnetic beads from cell extracts. Isolation of poly(A) RNA will mainly recover mRNA binding proteins, allowing for analysis of the cellular mRBPome. Stringent washing conditions are used to avoid isolation of unspecific binders.

***Note:*** As negative control for this protocol, we recommend the addition of an excess of polyadenylic acids (poly(A)) to the sample as a competitor. As an alternative, RNA is digested in the extract with an RNase. Optionally, a non-crosslinked sample has been used in a related protocol (Beckmann et al., 2015).

**Timing: 6–8 h**

14. Each experiment requires 500 μL of the yeast cell lysate/extract (10 mg/mL; ∼5 mg of protein lysate).
15. Control sample with poly(A) competitor: Add 20 μL of poly(A) (10 mg/mL) to 500 μL extract, briefly vortex sample and place on ice.

***Note:*** Optionally, an RNase digested control sample is prepared. Therefore, add 100 U RNase ONE to 500 μL of extract (∼ 5 mg protein lysate), briefly vortex and incubate for 2 h at 37°C.

16. Equilibrate one milligram of oligo[dT]_25_ Dynabeads in lysis buffer. Add 500 μL lysis buffer to the beads and vortex. Place tubes on the Dyna-Mag-2 magnet for at least 30 s and remove supernatant with a tip. Repeat three times and finally resuspend beads in 500 μL lysis buffer until use.
17. Remove lysis buffer from the pre-equilibrated oligo[dT]_25_ Dynabeads by placing the tubes on the Dyna-Mag-2 magnet. Add 500 μL of the extract and incubate the tubes on a shaker/rotating wheel for 10 min at room temperature.

***Note:*** We recommend using Eppendorf Protein LoBind microcentrifuge tubes for the poly(A) RIC protocol.

18. Retrieve the beads by placing the tubes in a Dyna-Mag-2 Magnet for at least 30 s and carefully remove the supernatant and transfer to a fresh low-bind tube.
19. Wash beads once with wash buffer A. Add 500 μL of pre-cooled wash buffer A on ice and gentle vortex for 10 s at room temperature.
20. Wash beads twice with wash buffer B. Add 500 μL of pre-cooled wash buffer B on ice and gentle vortex for 10 s at room temperature.

***Note:*** Repeated washing is required to remove unspecific binders from RNA, the beads and the tube wall. Wash buffer B also removes traces of Triton X-100, which may interfere with absorbance measurements and compromise elution efficiency (see step 25 below). Ensure complete removal of wash buffer at each step. We also recommend changing the tubes at least once during the procedure (e.g., between washes with buffer B).

21. Elute poly(A) RNA from beads. Add 60 μL of pre-heated elution buffer to the beads and incubate in a Thermoblock for 2 min at 80°C. Place tubes in a Dyna-Mag-2 Magnet and quickly transfer the eluate to a fresh tube.

***Note:*** Incubation at 80°C denatures the hybrid between poly(A) RNA and oligo[dT]_25_ beads, thereby releasing the poly(A) RNA into the solution (=eluate). Therefore, the eluate must be collected as quickly as possible to prevent re-association of eluted poly(A) RNA on beads. Elution from beads at 80°C follows the manufacturer’s suggestions. Other bead suppliers may use different temperatures.

***Note:*** An alternative for the elution is to release proteins via RNase digestion (see Beckmann et al., 2015).

22. Re-equilibrate the ‘eluted’ oligo[dT]_25_ Dynabeads in lysis buffer as in step 16.
23. Repeat step 17–22 twice by reapplying the supernatant to the oligo[dT]_25_ beads.
24. Combine the three sequential eluates (∼180 μL) and concentrate to ∼70 μL in a 0.5 mL Microcon-10 kD Centrifugal Filter Unit with Ultracel-10 membrane in a microfuge (14,000 × *g*, ∼30 min, 4°C).
25. Quantify eluted poly(A) RNA with a Nanodrop ND-2000 device.

***Note:*** We usually obtained ∼55 ng/ μL of poly(A) RNA. The 260/280 nm ratio should be at least 1.8:1. Lower ratios and an absorbance peak at 230 nm may relate to residual contamination from Triton X-100 in the eluate (see troubleshooting problem 2).

**Control step**: Successful poly(A) RNA/ mRNA isolation can be assessed with RT-PCR.

**Figure 2:**
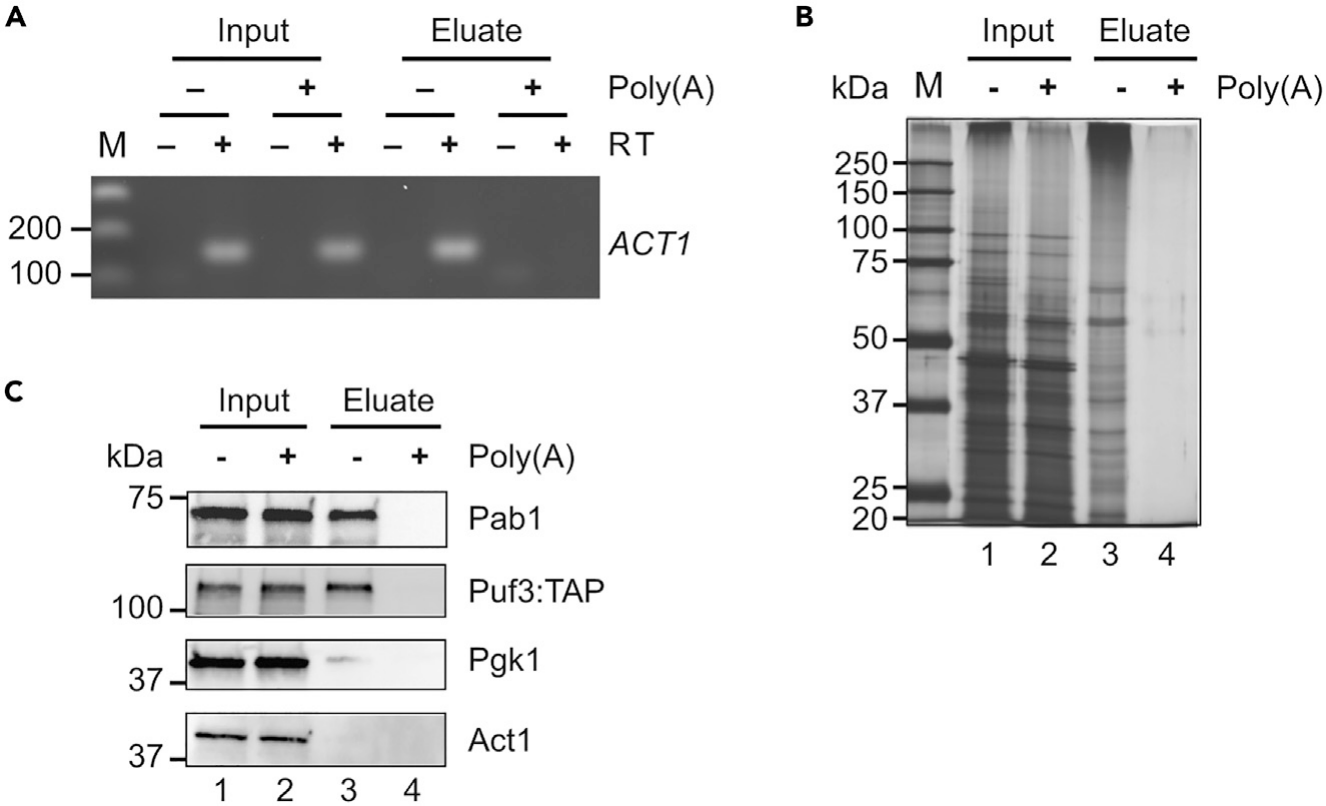
RNA and protein analysis of poly(A) RIC samples (A) RT-PCR on total RNA isolated from the extract (input) and RIC-eluates with *ACT1* specific primers. Products were resolved on a 2% agarose gel and stained with peqGREEN DNA/RNA dye. Poly(A) designates the addition of excess competitor poly(A). A control reaction without RT was included to monitor potential DNA contamination. M: molecular weight marker. (B) Silver stained PAA gel. Lanes 1–2 correspond to 0.05% of input extract and poly(A) treated control samples; lanes 3–4, 10% of eluates from poly(A) mRNA isolation. A Marker (M) with molecular weights (MW) in kilodaltons (KDa) is indicated to the left. (C) Immunoblot analysis to monitor the indicated proteins in 0.1% of the inputs (lanes 1–2) and 40% of the RIC eluates (lanes 3–4). Pab1, poly(A) binding protein 1; Puf3:TAP, tandem affinity purification-tagged Pumilio family protein; Pgk1, 3-phosphoglycerate kinase (a non-conventional RBP); Act1, actin (non-RNA binding control protein). Poly(A) designates the addition of excess competitor poly(A). MW is indicated to the left.

***Note:*** Poly(A) RNAs captured from negative control samples, either through poly(A) competition or RNase digestion should be included in the analysis to ensure that no mRNA is amplified in these samples (Figure 2A).

26. Detection of mRNAs in poly(A) eluates by reverse-transcription (RT) PCR (Figure 2A).
  a. 500 ng of input total RNA (Step 13.a) or 500 ng of eluted RNA (∼ 9.5 μL) are combined with a mixture of oligo[dT]_18_ and random hexamer primers for RT with the Transcriptor High Fidelity cDNA Synthesis Kit (Roche) following the manufacturer’s guidelines.
  b. PCR is conducted with 1 μL of complementary DNA (cDNA) with the following conditions:

**Table:** 

Steps	Temperature	Time	Cycles
Initial Denaturation	94°C	5 min	1
Denaturation	94°C	30 s	25–35 cycles
Annealing	57°C	30 s	
Extension	72°C	40 min	
Final extension	72°C	8 min	1
Hold	4°C	Forever
  c. PCR products are visualized on a 1.5% agarose gel stained with peqGREEN RNA/DNA dye.

### Detection of RNA-binding proteins: Silver stained gels and immunoblots

In this step, proteins bound to poly(A) RNAs are electrophoresed on SDS-polyacrylamide (PAA) gels and stained with silver (Figure 2B); and specific proteins are detected by immunoblot analysis (Figure 2C).

***Note:*** It is important to analyze proteins in the extract and RIC eluates. A silver stained PAA gel can ensure that the protein composition is different in the total extract, RIC eluates and negative control samples. Immunoblot analysis is used to monitor the presence of RBPs in the RIC eluates and not in the negative controls. These RBPs include the poly(A)-binding protein 1 (Pab1), which is known to be involved in regulating mRNAs fate and stability (Amrani et al., 1997; Sachs et al., 1987), and the PUmilio-homology domain Family 3 protein (Puf3), known to be abundantly associated with mRNAs to be involved in their localization and decay (Olivas and Parker, 2000; Gerber et al., 2004). A non-RBP can act as a negative control (e.g., actin1). These tests indicate whether mRBPome isolation has been successful.

**Timing: 2 days**

27. Analyze the protein complexity with a silver-stained PAA gel (Figure 2B).
  a. Resolve 0.05% of the input (=extract/lysate) and at least 10% of the eluates on a 4–15% Mini-PROTEAN® TGX™ Precast SDS-PAA gel.
  b. Run the PAA-gel at 120 V for 90 min in the appropriated electrophoresis chamber. Regularly check the progress of the sample migration.
  c. Stain gel with ProteoSilver™ Plus Silver Stain Kit, following the manufacturer’s guidelines.

***Note:*** One lane of the gel should contain a protein ladder as a molecular weight marker (*e.g.* Precision Plus Protein Dual Color Standards). We recommend loading up to 3 μL of a 1:10 dilution of the protein ladder to avoid an overstaining.

28. Identify specific proteins with immunoblots (Figure 2C).
  a. Resolve 0.1% of the input and at least 10% of the eluate on a 4%–15% Mini-PROTEAN® TGX™ Precast SDS-PAA Protein Gels.
  b. Run the gel for 90 min at 120 V.
  c. Cut a gel-sized polyvinylidene difluoride (PVDF) membrane and activate it with 100% methanol for 2 min.
  d. Soak the gel, filter papers, and PVDF membranes in the appropriate transfer buffer for 2 min at room temperature.
  e. Transfer proteins from the PAA gel to PVDF membranes with a Trans-Blot® Turbo™ Transfer System (or equivalent).
    ***Note:*** Voltage and length of transfer may vary depending on the molecular weight of the protein of interest. Proteins with high molecular weight (>200 kDa) need longer transfer time.
    ***Note:*** We recommend checking for transfer of proteins by staining the membrane with Ponceau-S solution according to manufacturer’s instructions. We also recommend loading a pre-stained protein marker (e.g. Precision Plus Protein Dual Color Standards).
  f. Block membrane in 1× PBS-0.1% Tween-20 (PTST) containing 5% low fat milk for 1 h at room temperature.
  g. Probe membrane with respective primary antibodies (e.g., anti-Pab1 (1:5,000), anti-Pgk1 (1:5,000), anti-actin (1:2,500), PAP reagent (1:5,000)) diluted in PTST containing 2% low fat milk, for 1 h at room temperature with constant shaking.
  h. Wash the membrane 3 times in PBST for 10 min each at room temperature with constant shaking.
  i. Add the corresponding horseradish peroxidase (HRP)-coupled secondary antibodies (1:5,000) diluted in PTST containing 2% low fat milk for 1 h at room temperature with constant shaking.
  j. Wash the membrane 3 times in PBST for 10 min each at room temperature with constant shaking.
  k. Develop membrane with the Immobilon Western Chemiluminescent HRP Substrate, following the manufacturer’s guidelines.
  l. Take a picture of the chemiluminescent signals with standard gel imaging system.

***Note:*** Immunoblotting has been described in detail (Litovchick, 2020; Burckhardt et al., 2021). Of note, proteins (i.e. Pab1) may shift in the gel to a higher molecular weight due to crosslinks to RNA.

***Note:*** Use of fluorescently labeled antibodies and detection with a fluorescence scanner may be considered to obtain a more quantitative read-out.

## Expected outcomes

The herein described processing of 500 mL yeast cells yields ∼12 mg of protein in the extract and ∼7.5 μg in poly(A) RIC eluates. Thus, about 0.06% of protein from the total protein extracts is usually recovered with this RIC protocol. The amount of captured proteins is sufficient to proceed with label- free mass spectrometry (MS) analysis, allowing for identification of the yeast mRBPome under different stress conditions (Matia-González et al., 2015, 2021). Of note, this protocol is not biased towards the identification of canonical RNA-binding proteins that contain characteristic RNA-binding domains, as our MS data revealed multiple unconventional RBPs, lacking such domains including many metabolic enzymes (Matia-González et al., 2021).

A successful poly(A) RIC enables the monitoring of particular mRNAs, such as *ACT1* mRNA, in the RIC eluates directly by RT-PCR (Figure 2A), adding the input (total lysate) as reference. Poly(A) RNAs captured from negative control samples, either through poly(A) competition or RNase digestion should be included in the analysis to ensure that no mRNA is amplified in these samples (Figure 2A). In addition, a different protein pattern in the RIC eluate when compared with input extract is observed as result of the poly(A) RIC. Hardly any proteins should be detected in the poly(A) competition control experiment (Figure 2B). Of note, using different type of negative controls than the one used in this protocol, such as non-crosslinked samples, might result in the detection of some protein bands in the eluates (troubleshooting problem 3). Well known RBPs, such as Pab1 or Puf3, as well as non-conventional RBPs including the metabolic enzyme Pgk1, can be detected in the RIC eluate by immunoblot (troubleshooting problem 4). Conversely, proteins not expected to bind poly(A) RNAs such as actin should not be detectable in the eluate fraction (Figure 2C).

By applying this protocol, we have defined the mRBPome in the budding yeast *Saccharomyces cerevisiae* (Matia-González et al., 2015) comprised of 765 proteins. Moreover, we applied this protocol to define the mRBPome under oxidative stress conditions, identifying 257 proteins that differentially associate with poly(A) RNA (Matia-González et al., 2021). This protocol also helped in revealing a rearrangement of the poly(A) RNA association upon oxidative stress of several metabolic pathways, specially, the ones implicated in carbon metabolism. Furthermore, we found that the RNA-binding capacity of these enzymes was paralogue specific.

## Limitations

This protocol is based on capturing poly(A) RNAs from cells, a major component of it comprising mRNAs. Hence, it is less suitable for identification of RBPs that preferentially interact with other RNA species that lack a poly(A) tail, such as rRNAs and tRNAs. Furthermore, the protocol is based on UV-irradiation of cells to crosslink RNA protein interactions *in vivo*. The efficiency UV-based crosslinking is relatively low (∼5%) and shows some preference for uridine-rich single-stranded RNA regions, possibly adding a bias towards single-stranded RNA-binding proteins. In addition, we wish to note that UV-irradiation can also induce protein-protein and protein-DNA crosslinks - albeit at lower efficiency – but may lead to false positives through indirect interactors (Gerber, 2021). Other types of crosslinking, such as the photoactivatable ribonucleoside-crosslinking (PAR-CL) (Shchepachev et al., 2019) or the formaldehyde-based crosslinking (FA-CL) (Na et al., 2021) may constitute suitable alternatives.

## Troubleshooting

### Problem 1

RNA degradation

### Potential solution

To avoid RNA degradation during UV-irradiation and lysate preparation, it is very important to keep cells on ice (step 3e). UV-light can directly damage RNA and a careful check of the settings and prior testing of the UV-crosslinker is recommended. Since the suspension absorbs the energy from UV-light and warms-up, we recommend keeping cells on ice for 2 min between three UV exposures (400 mJ each) (Figure 1B). After UV-irradiation, immediately collect the cells at 4°C and snap freeze in liquid nitrogen or proceed immediately with the preparation of the extract. Add sufficient amounts of RNase inhibitors to the lysis buffer. If degradation occurs during poly(A) RIC, supplement the wash buffers with 1 U/mL of RNasin.

### Problem 2

UV-spectra of eluted RNA with a 260/280 nm ratio of less than 1.8 alongside peak absorbance at 230 nm (step 25).

### Potential solution

The eluate may still contain some detergents supplied in lysis and wash buffer A (Triton X-100 absorbs in the range of 230 nm and at 280 nm). This can be solved by a quick spin of tubes in a microfuge (1,000 × *g*) and careful removal of residual wash buffer B (step 20) with a pipette.

### Problem 3

Detection of proteins in the eluate of ne*g*ative controls (steps 26 and 27).

### Potential solution

The detection of non-specific RNA-protein interaction in poly(A) competitor or in non-crosslinked samples (data not shown) is due to non-specific protein-protein interactions or non-covalent binding of protein to the RNA. This has been solved by increasing the stringency of the washes during the poly(A) RNA pull down through addition of 0.2% lithium dodecyl sulfate (LiDS) to wash buffer A (step 19).

### Problem 4

Absence of signal for a determined protein in the eluate fraction monitored by immunoblots (step 27).

### Potential solution

The detection of proteins in the poly(A) RNA eluate with immunoblots depends on a variety of instances, ranging from biological factors like protein and mRNA target levels and their affinities to technical variables, such as transfer of protein to membrane and quality of antibodies. Therefore, resolving 10% of the RIC eluate may not be sufficient for reliable detection and requires loading of a higher fraction of the eluate (up to 40%). To monitor the success of the experiment we recommend using a positive control such as Pab1 - a highly expressed protein binding to the poly(A) tail of mRNAs. However, in many cases no suitable antibodies for detection of a specific protein may be available. In that case, strains bearing TAP/GFP-tagged proteins can be used, allowing detection of tagged-proteins with highly sensitive commercially available reagents (e.g., Puf3-TAP, Figure 2C). Finally, we wish to note that besides the input and eluate, samples can also be taken from the supernatant (i.e., extract after incubation with oligo[dT]_25_ beads) and wash fractions to follow the protein under consideration during the entire procedure.

## Resource availability

### Lead contact

Further information and requests for resources and reagents should be directed to and will be fulfilled by the lead contact, André P. Gerber (a.gerber@surrey.ac.uk).

### Materials availability

This study did not generate new unique reagents.

## Data Availability

This study did not generate/analyze datasets/code*.*
